# Estimating the Burden of Illness Related to Genital Warts in Russia: A Cross-Sectional Study

**DOI:** 10.36469/jheor.2020.17246

**Published:** 2020-10-07

**Authors:** Vera N. Prilepskaya, Mikhail Gomberg, Smita Kothari, Karen Yee, Amit Kulkarni, Suzanne M. Garland, Anna R. Giuliano

**Affiliations:** 1 Ministry of Healthcare of the Russian Federation, Research Center for Obstetrics, Gynecology, and Perinatology, Moscow, Russia; 2 Moscow Scientific and Practical Center for Dermatovenerology and Cosmetology, Moscow, Russia; 3 Merck & Co. Inc., Kenilworth, NJ, USA; 4 Cubist Pharmaceuticals, Lexington, MA, USA; 5 The Royal Women’s Hospital, Murdoch Childrens Research Institute, Department of Obstetrics and Gynecology, University of Melbourne, Parkville, Victoria, Australia; 6 Center for Infection Research in Cancer (CIRC) at Moffitt Cancer Center, Tampa, FL, USA

**Keywords:** Human Papillomavirus (HPV), Vaccine, Disease Prevention, Infectious Diseases, Condyloma

## Abstract

**Background:**

Human papillomavirus (HPV) infections are the etiologic agents of genital warts (GW). HPV is one of the most frequent sexually transmitted viral infections, and nearly 65% of individuals with partners who have GW also develop GW. In Russia, as in many other countries, overall GW prevalence data are scarce. Given the lack of Russian data, our study estimated GW prevalence in physician practices and GW-related health care resource use in Russia among male and female patients aged 18–60 years.

**Methods:**

Russian physicians recorded daily patient logs for a two-week period and conducted a 30-minute survey to estimate GW prevalence and related resource use between January and June 2012. Age, gender, and GW diagnosis status was recorded. Prevalence was obtained for each physician and calculated into a single estimate across all physician types. Overall prevalence estimate and 95% confidence interval were weighted by the estimated number of physicians in each specialty and the proportion of total patients visiting each specialist type. Health care resource use was reported and compared among different physician specialties.

**Results:**

The overall GW prevalence estimate was 9162 cases per 100 000 for male and female patients aged 18–60 years, with 9917 for obstetrician/gynecologists (OB/GYN), 8298 for urologists (URO), and 7833 for dermatologists (DERM). For males, GW prevalence was 8769 cases per 100 000, with the highest prevalence in the 30–34 age group. In females, GW prevalence was 9304 cases per 100 000, with the highest prevalence in the 18–24 age group. Among overall existing GW cases, 63.1% were recurrent and 34.2% were resistant. For all patients in our study, GW prevalence was higher in females. Male patients had the highest prevalence for those aged 30–34 years, and female patients for those aged 18–24 years. These results are consistent with data reported in other countries. Study limitations include estimates and results representative of the urban population of Russia. Despite its limitations, this study provides a GW prevalence estimate in Russia not previously available.

**Conclusions:**

GW is a significant public health concern in Russia, and the GW prevalence was higher in female patients compared to male patients.

## BACKGROUND

Human papillomavirus (HPV) infections are the etiologic agents of GW. More than 130 different types of the virus have been identified and divided into two groups according to epidemiological association with cervical cancer.^1^ The low-grade HPV group includes types 6 and 11, which are estimated to cause approximately 90% of GW cases.^1^,^2^ The high-risk HPV group, including types 16 and 18, causes precancerous lesions such as cervical intraepithelial neoplasia, cervical cancer, and anogenital cancer.^3^

HPV is one of the most frequent sexually transmitted viral infections, and nearly 65% of individuals with partners who have GW also develop GW.^4^,^5^ An estimated 6.2 million persons are newly infected every year in the United States alone, but most infections are asymptomatic or subclinical and become undetectable over time.^6^

Data on national GW incidence by country is limited, and prevalence estimates by country range widely, from 1.4% (Spain) to 25.6% (Nigeria).^7^–^9^ In Russia, as in many other countries, most available literature focuses on the oncogenic HPV type, and overall GW prevalence data are scarce. Bogdanova et al. evaluated the epidemiology of viral STIs from 2000 to 2011 in the Russian Federation and concluded that the total rates of genital herpes simplex virus infection remained constant (mean rate 19.8±1.4 per 100 000) while the total HPV infection rate during the same period increased from 27.4 to 29.2 per 100 000, with maximum reported rates of 34.7 in 2009.^10^

The large economic burden of GW treatment and management weighs heavily on a health care system. With rates of newly diagnosed GW cases increasing, the economic burden is also likely to increase, as even self-resolved GW cases can recur and in some cases are resistant to treatment. Currently available GW treatments include patient-applied (home-based) chemical treatments (podofilox, imiquimod), provider-administered (office-based) chemical treatments (podophyllin, trichloroacetic acid, interferon), and ablative treatments (cryotherapy, surgical removal, laser treatment).^11^

Although this study looks at the prevalence and healthcare utilization of GW within the Russian population, it can be generally noted that an increasing prevalence of GW incurs increased healthcare utilization resulting in higher economic cost. A study that looked at incidence and economic burden of GW in Korea showed that the total cost of outpatient clinics in 2015 was approximately $9.3 million.^12^ Another study that looked at GW in the United Kingdom estimated over 220 000 cases of GW in 2012, resulting in £58.44 million with £265 per patient.^13^ In 2008, Hillemanns et al. estimated overall third-party payer GW costs at €49.0 million and total societal costs at €54.1 million in Germany.^14^ In Spain, Castellsagué et al. estimated overall third-party payer GW costs at €47 million and total societal costs at €59.6 million in 2009.^7^ A recent study assessing the incidence and economic burden on the US commercially insured population reported estimated costs at $760 per 1000 individuals in the general population in 2004, with total costs exceeding $220 million.^15^ An Australian study showed an annual incidence of 2.19 cases of GW per 1000 Australians (95% confidence interval [CI]: 1.88 to 2.49). In addition, the estimated cost of managing GW annually was over US$14 million, with an estimated cost per treated case of US$251 for men and US$386 for women.^16^ To date, little research has been conducted to analyze GW incidence and prevalence in Russia. Data available in Russia focuses predominantly on cervical cancer. The country-specific, overall high-risk HPV frequency in republics of the former Soviet Union was estimated at 33.4%, and HPV 16 was found to be the most prevalent type in Russia (205/1967 women).^17^ A study of female patients aged >30 in St. Petersburg indicated that high-risk HPV was present in 13% of the study population.^18^ As such, the likelihood of GW caused by HPV and the significant economic impact on society may be higher than estimated. Given the lack of available data in Russia, the current study was designed to estimate GW prevalence in physician practices and GW-related health care resource use in Russia among male and female patients aged 18–60 years.

## METHODS

### Study Design

This was a cross-sectional study conducted through physician surveys in major federal districts in Russia, including Central (Moscow, Reutov, Mozhaysk, Yaroslavl, Belgorod, Voronezh, Lipetsk), Privolzhskiy (Penza, Saransk, Ulyanovsk, Orenburg, Izhevsk), Sibirskiy/Ural (Novosibirsk, Chelyabinsk, Tyumen), and Northwest (Baltiysk, Kaliningrad, St. Petersburg).

The study period was from January through June 2012. Ethical approval was obtained from an accredited external Institutional Review Board (IRB) for those sites not covered by their own internal IRB. The ethical conduct of this study was performed in accordance with the Declaration of Helsinki and the principles of Good Clinical Practices.^19^

#### Study Instruments

Two study instruments, including a physician survey and a two-week daily log, were used. The 30-minute physician survey posed questions related to resource use as part of the usual course of diagnosis, treatment, and follow-up care (inpatient, outpatient) for typical GW patients in participating physicians’ practices. The survey was completed by physicians who attended to and treated GW patients. The survey also included questions related to practice referral patterns, from general practice physicians to specialists as well as between specialists.

The physician two-week daily log recorded the number of all-cause, newly diagnosed, and existing GW patients (ie, GW was the primary reason for the visit or GW was diagnosed during the visit), patients retained versus referred to other specialists for treatment, and patient age and gender information for all patients seen.

#### Inclusion and Exclusion Criteria

Participating physicians were identified through a Clinical Research Organization representative. The investigator list was compiled before study initiation and based on a database of investigators who participated in other clinical trials, an investigator list of those contacted during feasibility phases of other clinical studies, and physician and hospital contact information from open sources. The list contained an equal number of physicians in all three specialties from different cities.

Physicians included in this study (a) were specialists (obstetrician/gynecologists [OB/GYN], urologists [URO], and dermatologists [DERM] with 2–35 years of practice experience) that provided informed consent to participate, (b) devoted ≥30% of their time to treating patients for outpatient visits three or more work days per week (as opposed to inpatient surgeries, teaching or other activities) and spent 2 or more work days seeing patients for outpatient visits, (c) treated ≥50 patients for outpatient visits in a typical week, and (d) treated ≥50% of patients aged 18–60 years for outpatient visits.

Practice settings of participating physicians included private office/clinic, private hospital, and public health care center/clinic or hospital (including university and military hospitals). There were no exclusion criteria for physicians participating in the study.

#### Prevalence and Health Care Resource Use

GW prevalence was estimated from the physician daily logs for patients seen over a two-week period. The number of newly diagnosed and existing GW cases was captured during consultations recorded in the physician daily logs. GW prevalence was estimated using a stratified estimator based on weights calculated to adjust for the difference between the observed number of physicians in each specialty and the (externally) estimated number of physicians in each specialty, at the national level.

The GW prevalence in physician practices was calculated using the number of newly diagnosed or existing GW cases observed, divided by the total number of patients seen during the two-week study period. The prevalence was calculated for overall patients, each physician specialty type, and patient age group and gender. Prevalence by specialty from the study sample was then adjusted to the national level to provide one national prevalence estimate across all specialties.

The prevalence of newly diagnosed and existing cases was estimated using a stratified estimator based on weights calculated to adjust for the difference between the observed and estimated number of physicians in each specialty at the national level. According to estimates by Merck Sharp & Dohme (MSD), a total of 24 000 OB/GYN, 9600 DERM, and 6000 URO practice on the national level in Russia.

Data from the two-week daily logs were also used to estimate GW prevalence for patients aged 18–60 years in four federal districts in Russia (Central, Privolzhskiy, Sibirskiy/Ural, and Northwest). Population estimates of physicians per specialty group (as per MSD) were divided equally among the regions.

Referral patterns and resource use for GW patients were captured through a 30-minute face-to-face physician survey during the study period from January to June 2012. The survey included questions related to resource use, treatment (in-office treatments and procedures, in-office or at-home topical treatments), and follow-up care (office visits, emergency room visits, hospitalizations) for typical GW patients in the practice as part of the standard course of diagnosis. Survey questions were designed to determine patient referral patterns from general practice physicians to specialists as well as between specialists. Referral patterns were assessed using the physician survey, which included the percentage of patients referred by other physicians as well as those consulted directly with OB/GYN, DERM, or URO.

#### Statistical Analysis

All data analyses and summaries were performed using SAS^®^ Version 9.2. All study outcomes were summarized descriptively. Categorical data were summarized using counts and percentages, and continuous data were summarized using number of observations and mean values.

Prevalence was stratified by patient age group, patient gender, and physician specialty. Number, mean, and 95% CI were reported. The number and percentage of newly diagnosed and existing GW patients were reported by physician specialty type. Recurrent and resistant cases for existing GW patients were specified.

Referral patterns for GW patients were reported descriptively. The number and mean percentage of patients who directly consulted with, or were referred by, each physician specialty type were reported. Health care resource use was reported and compared among physician specialty types.

## RESULTS

### Prevalence

A total of 103 physicians completed the two-week daily log, including 28 OB/GYN, 40 URO, and 35 DERM. Information was recorded for approximately 15 961 patients, with 1369 GW cases observed in patients aged 18–60 years. When combining the MSD estimates of practicing physicians with the average number of patients reported on the two-week daily log, an overall patient population seen during the two-week daily log period was estimated at 3 811 714 patients for OB/GYN, 842 400 for URO, and 1 617 737 for DERM.

Based on these data, the overall weighted estimated GW prevalence was calculated at 9.2% (95% CI: 8.3%–10.0%). For each specialty group, the unweighted estimated GW prevalence was 9.9% for OB/GYN, 8.3% for URO, and 7.8% for DERM (Table 1).

**Figure 1:**
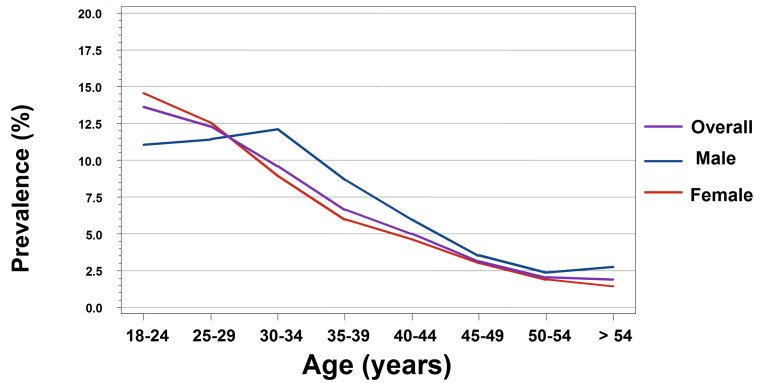
GW Prevalence by Age Group and by Gender for Patients Age 18–60 Years (Participating Physicians for Two-Week Daily Log)

**Figure 2:**
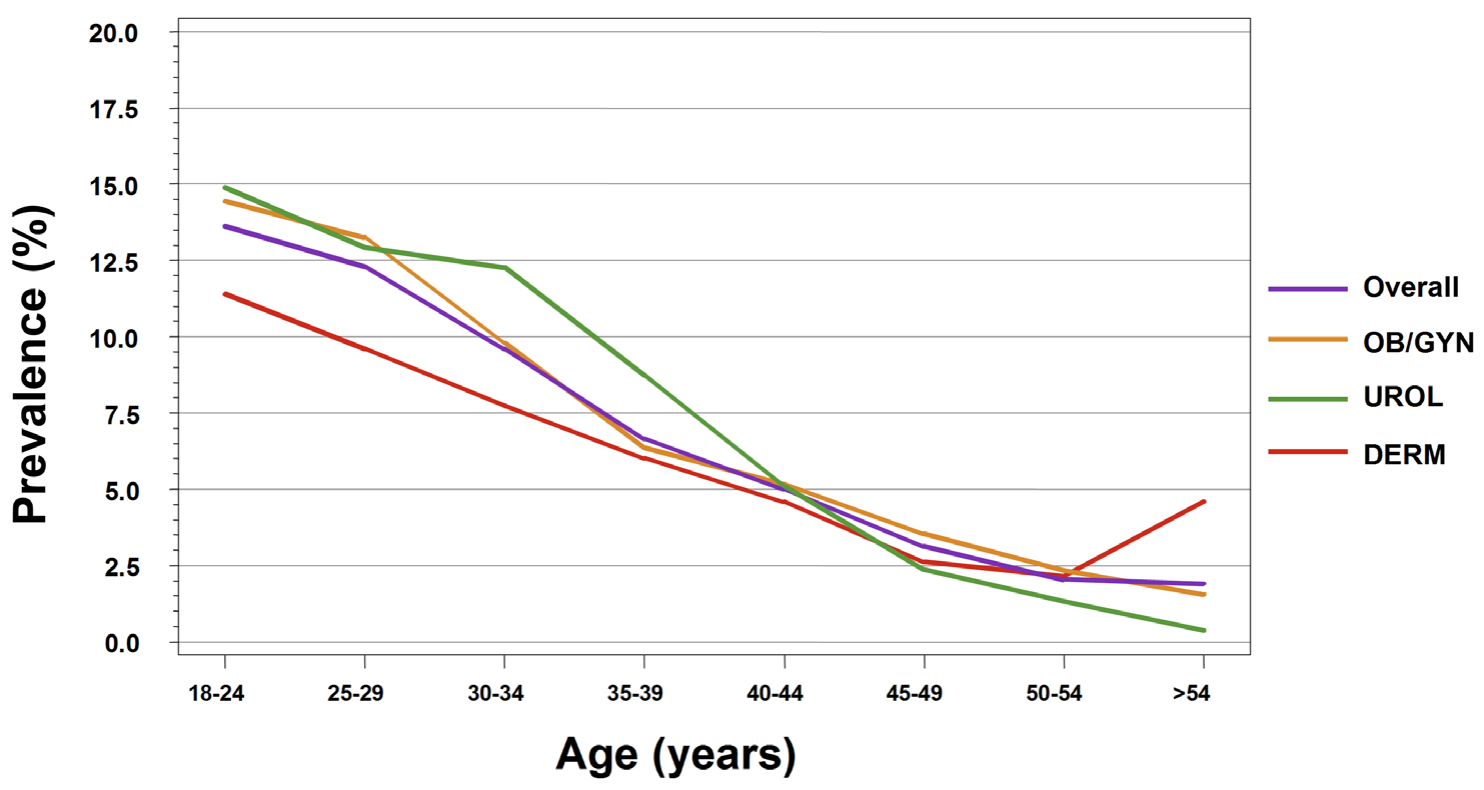
GW Prevalence by Age Group and Physician Specialty for Patients Aged 18–60 Years (Participating Physicians for Two-Week Daily Log)

A total of 15 914 patients were reported in the two-week daily logs. Among the 7513 male patients reported in the two-week daily logs, a total of 673 GW cases were reported, resulting in a weighted total observed GW prevalence of 8.8% (95% CI: 7.9%–9.7%). For male patients, the highest prevalence was calculated for those aged 30–34 years (12.1%), followed by 25–29 years (11.4%) and 18–24 years (11.0%) (Figure 1). For each specialty group, the unweighted estimated GW prevalence for male patients was 9.5% for URO and 8.3% for DERM (Figure 2).

Among the 8401 total female patients included in the two-week daily logs, a total of 695 GW cases were reported, resulting in a weighted total observed GW prevalence of 9.3% (95% CI: 8.4%–10.2%). The highest prevalence was calculated for those aged 18–24 years (14.5%), followed by 25–29 years (12.6%) (Figure 1). For each specialty group, the unweighted estimated GW prevalence for female patients was 9.9% for OB/GYN, 5.0% for URO, and 7.3% for DERM (Figure 2).

For male and female patients seeing all physician specialties, the estimated GW prevalence was the highest for patients aged 18–24 years (OB/GYN: 14.4%; URO: 14.9%; DERM: 11.4%). GW prevalence decreased as patient age increased (Figure 2).

Prevalence stratified by region is presented in Table 2. For all patients, the unweighted estimated GW prevalence was the highest in the Sibirskiy/Ural federal district (10.0%), followed by the Northwest (8.9%) and Central federal districts (8.8%). The lowest prevalence was noted in the Privolzhskiy federal district (6.4%).

For male patients, the unweighted estimated GW prevalence was 8.8% in the Central federal district, 8.0% in Privolzhskiy, 14.4% in Sibirskiy/Ural, and 8.9% in the Northwest federal district. For female patients, the unweighted estimated GW prevalence was 8.9% in the Central federal district, 4.9% in Privolzhskiy, 6.9% in Sibirskiy/Ural, and 8.9% in the Northwest federal district.

Overall, 941 newly diagnosed and 407 existing GW cases were identified. Among existing GW cases, 63.1% were recurrent and 34.2% were resistant. The percentage of patients with a resistant form of GW ranged from 19.4% in URO to 46.5% in DERM (Table 3).

For an in-depth view of gender-stratified referral patterns and health care resource use, please see the Supplementary Material, which includes a Supplemental Table and Supplemental Figures.

## DISCUSSIONS

This multicenter observational study was conducted in Russia to assess GW prevalence in physician practices and GW-related health care resource use among male and female patients aged 18–60 years.

At the Russian national level, the current study estimated GW prevalence to be 8.8% in male and 9.3% in female patients, which is higher compared to estimates reported in recent literature for other countries.^20^,^21^ For instance, the US National Health and Nutrition Examination Survey found that from 1999 through 2004, 5.6% of survey respondents (aged 18–59 years) self-reported a GW diagnosis.^22^ The percentage was higher for women (7.2%; 95% CI: 6.2%–8.4%) compared to men (4%; 95% CI: 3.2%–5.0%). Although our study did not examine the reasons for the high rate of GW in Russia, several factors could be leading to this high rate. One factor to consider is the knowledge, attitude, and practices surrounding STIs in Russia. A study by Lan et al. observed an association between a high rate of alcohol consumption and an increased risk of STIs, such as HIV, in Russia.^23^ Other factors could be healthcare accessibility for patients with STIs and inadequate health education on STIs prevention and transmission.

Results from a systematic review of GW incidence and prevalence conducted in four Nordic European countries showed a wide range of prevalence in the self-reported history of GW.^24^ In surveys of general adult populations, 0.36% (Slovenia, sexually active, aged 18–49 years) to 12.0% (Iceland, aged 18–45 years) of females reported a lifetime history of GW.^5^ In male populations, 0.27% reported a history of GW in Slovenia from November 2004 to June 2005, and 3.6% to 7.9% reported in Australia, Denmark, the United Kingdom, and the United States.^5^,^22^,^25^–^27^

For all patients included in the current study, GW prevalence was higher in females than in males (9.3% vs 8.8%). When examining a single gender group, male patients had the highest prevalence at those aged 30–34 years, and female patients at 18–24 years, both followed by patients aged 25–29 years. These results are consistent with data reported in in other countries. In a study conducted in the United States, GW incidence was highest among female patients aged 20–24 years (4.6 cases per 1000) and males aged 25–29 (2.7 cases per 10 000).^21^ Additionally, in an Australian study population, GW incidence peaked in women aged 20–24 years (8.61 cases per 1000) and in men ages 25–29 years (7.40 cases per 1000).^28^ In Canada, GW prevalence was the highest among females aged 20–24 years (3.88 cases per 1000), whereas prevalence peaked at 25–29 years in men (3.69 cases per 1000).^29^ Unlike results from the current study, GW prevalence between 1998 and 2006 in Canada was always higher in male than in female patients.

GW prevalence stratified by region showed the highest prevalence to be in the Sibirskiy/Ural federal district and the lowest in Privolzhskiy. The Sibirskiy/Ural federal district includes the cities of Novosibirsk, Chelyabinsk, and Tyumen, while the Privolzhskiy federal district includes Penza, Saransk, Ulyanovsk, Orenburg, and Izhevsk. These are the first available data comparing GW prevalence across regions of Russia.

The most recommended and effective treatment options for GW treatment are podofilox, imiquimod, surgical excision, and cryotherapy.^30^ In the current study, the treatment pattern reported suggested that podofilox was the most frequently used therapy in male patients. Other topical medications (cryopharma spray, curiosin, cycloferon, epigen, genferon levomekol, panavir, pheresolum, pimafucort, rebif, resorcinol, solcoderm, tricresol, triderm, lactic acid, salicylic acid) were most frequently used to treat female patients. In terms of in-office treatments and procedures, a basic office visit was more frequently conducted by participating physicians. In both male and female patients, a basic office visit was followed by electrosurgery. However, a study that looked at effect of the HPV vaccine in Australia saw a significant yearly decrease in the diagnosis and management rate of GW among women of vaccine-eligible age, indicating a decrease in the development of GW among this population.^31^

Although there are limited data on GW in Russia, the burden of cervical cancer in the country indicates alarming rates in certain regions. In fact, incidence and mortality from cervical cancer are substantially higher in Eastern than in Western European countries mainly due to the lack of effective screening programs.^18^,^32^

In Russia, more than 6000 women succumb to cervical cancer annually, accounting for approximately 4.6% of all cancer-related deaths among Russian women.^18^ No organized screening programs exist in Russia, and cervical cancer prevention is based on opportunistic screening with low coverage. Screening is also poorly standardized without quality-assured cytology and colposcopy.^18^

The current study has several limitations. The national prevalence estimates were based on the physician population available from medical societies and may not include all practicing physicians in Russia. In addition, estimates and results in this study are representative of the urban population of Russia.

GW patients who did not seek health care were not included, which may underestimate the true prevalence in Russia since weighting was applied.

Potential bias related to the information source, based on a physician survey for the estimation of health care resource use, may exist since bias in recall estimations by physicians can be difficult to control for.

## CONCLUSIONS

GW is a significant public health concern. The aim of this study was to estimate the burden of GW as well as the GW-related health care resource use for male and female patients aged 18–60 years in Russia. The overall GW prevalence in Russia was estimated at 9.2%, with higher prevalence in female compared to male patients. GW prevalence was highest in female patients aged 18–24 and male patients aged 30–34 years.

OB/GYN and URO practices saw a high prevalence of GW patients. Visual examination was the most common diagnostic tool used by all physician specialists for both male and female patients. Despite its limitations, this study provides a GW prevalence estimate in Russia not previously available. In addition, we recommend further studies to evaluate the factors contributing to the high prevalence of GW in Russia.

## Supplementary Information



## Figures and Tables

**Table 1:** GW Prevalence for All Patients Age 18–60 Years (Participating Physicians for Two-Week Daily Log)

	Specialty Group			
	OB/GYN	URO	DERM	Overall
**Number of physicians in study sample**	28	40	35	.
**Number of patients reported on two-week daily log**	4447	5616	5898	15 961
**Mean N of patients reported on two-week daily log**	158.8	140.4	168.5	.
**Patient distribution in two-week daily log**	0.2786	0.3519	0.3695	1.0000
**Observed GW cases**	441	466	462	1369
**Sample prevalence (per 100 000 population)**	9916.798	8297.721	7833.164	8577.157
**Number of physicians at national level**^a^	24 000	6000	9600	.
**Number of patients at national level**	3 811 714	842 400	1 617 737	.
**Distribution of patients at national level**	0.6077	0.1343	0.2579	.
**Estimated prevalence (per 100 000 population)**^b^	9916.798	8297.721	7833.164	9161.888
**Standard error**^b^	448.2023	368.0916	349.8675	432.1853
**95% Confidence interval**^b^	(9038.321, 10 795.27)	(7576.261, 9019.180)	(7147.424, 8518.904)	(8314.805, 10 008.97)

Abbreviations: DERM, dermatologist; GW, genital warts; MSD, Merck Sharp & Dohme; OB/GYN, obstetrician/gynecologist; URO, urologist.

a Population estimates of the number of physicians per specialty group were provided by MSD.

b Unweighted estimates used within specialty groups and weighted estimates used for Overall.

**Table 2:** GW Prevalence for All Patients Age 18–60 Years by Region (Participating Physicians for Two-Week Daily Log)

	Region (by Federal District)				
	Central	Privolzhskiy	Sibirskiy/Ural	Northwest	Overall
**Number of physicians in study sample**	56	12	5	30	.
**Number of patients reported on two-week daily log**	8127	2102	719	5013	15 961
**Mean number of patients reported in two-week daily log**	145.1	175.2	143.8	167.1	.
**Patient distribution in two-week daily log**	0.5092	0.1317	0.0450	0.3141	1.0000
**Observed GW cases**	719	134	72	444	1369
**Sample prevalence (%)**	8.8471	6.3749	10.0139	8.8570	8.5772
**Number of physicians at national level**^a^	9150	9150	9150	9150	.
**Number of patients at national level**	1 327 894	1 602 775	1 315 770	1 528 965	.
**Patient distribution at national level**	0.2299	0.2775	0.2278	0.2647	.
**Estimated prevalence (%)**^b^	8.8471	6.3749	10.0139	8.8570	8.4294
**Standard error**^b^	0.3150	0.5329	1.1195	0.4013	0.6409
**95% Confidence interval**^b^	(8.2296, 9.4645)	(5.3305, 7.4193)	(7.8197, 12.2081)	(8.0704, 9.6435)	(7.1733, 9.6856)

Abbreviations: DERM, dermatologist; GW, genital warts; MSD, Merck Sharp & Dohme; OB/GYN, obstetrician/gynecologist; URO, urologist.

a Population estimates of the number of physicians per specialty group were provided by MSD and divided equally among regions.

b Unweighted estimates used for specialty groups and Overall.

**Table 3:** GW Case Description by Specialty in Russia

Cases	OB/GYN	URO	DERM	Overall
**New or existing GW**				
New case	261 (59.2%)	327 (70.2%)	353 (76.4%)	941 (68.7%)
Existing case	174 (9.5%)	134 (28.8%)	99 (21.4%)	407 (29.7%)
Missing	6 (1.4%)	5 (1.1%)	10 (2.2%)	21 (1.5%)
**Existing cases**				
Recurrent	105 (60.3%)	105 (78.4%)	47 (47.5%)	257 (63.1%)
Recalcitrant to treatment	67 (38.5%)	26 (19.4%)	46 (46.5%)	139 (34.2%)
Missing	2 (1.1%)	3 (2.2%)	6 (6.1%)	11 (2.7%)

Abbreviations: DERM, dermatologist; GW, genital warts; OB/GYN, obstetrician/gynecologist; URO, urologist.

New Case: GW case not diagnosed previously by self or another physician.

Existing Case: GW case that was diagnosed previously by self or another physician.

Recurrent Case: Previous GW episodes had resolved with treatment.

Resistant Case: Previous GW episodes had not resolved with treatment.

## References

[1] Haupt RM, Sings HL (2011). The efficacy and safety of the quadrivalent human papillomavirus 6/11/16/18 vaccine gardasil. J Adolescent Health.

[2] Garland SM, Steben M, Sings HL (2009). Natural history of genital warts: analysis of the placebo arm of 2 randomized phase III trials of a quadrivalent human papillomavirus (types 6, 11, 16, and 18) vaccine. J Infect Dis.

[3] Kim MA, Oh JK, Kim BW (2012). Prevalence and seroprevalence of low-risk human papillomavirus in Korean women. J Korean Mel Sci.

[4] Patel H, Wagner M, Singhal P, Kothari S (2013). Systematic review of the incidence and prevalence of genital warts. BMC Infect Dis.

[5] Pirotta MV, Stein AN, Fairley CK (2009). Patterns of treatment of external genital warts in Australian sexual health clinics. Sex Transm Dis.

[6] Dunne EF, Nielson CM, Stone KM, Markowitz LE, Giuliano AR (2006). Prevalence of HPV infection among men: a systematic review of the literature. J Infect Dis.

[7] Castellsagué X, Cohet C, Puig-Tintoré LM (2009). Epidemiology and cost of treatment of genital warts in Spain. Eur J Public Health.

[8] Graziottin A, Serafini A (2009). HPV infection in women: psychosexual impact of genital warts and intraepithelial lesions. J Sex Med.

[9] Clifford GM, Gallus S, Herrero R (2005). Worldwide distribution of human papillomavirus types in cytologically normal women in the International Agency for Research on Cancer HPV prevalence surveys: a pooled analysis. Lancet.

[10] Bogdanova E, Melekhina L (2013). P3.033 Epidemiology of viral STIs in 2000–2011 in Russian Federation. Sex Transm Infect.

[11] Centers for Disease Control and Prevention 2015 Sexually Transmitted Diseases Treatment Guidelines: Anogenital Warts.

[12] Park YJ, Kim JM, Lee BR, Kim TH, Lee EG (2018). Annual prevalence and economic burden of genital warts in Korea: Health Insurance Review and Assessment (HIRA) service data from 2007 to 2015. Epidemiol Infect.

[13] Coles VA, Chapman R, Lanitis T, Carroll SM (2016). The costs of managing genital warts in the UK by devolved nation: England, Scotland, Wales and Northern Ireland. Int J STD AIDS.

[14] Hillemanns P, Breugelmans JG, Gieseking F (2008). Estimation of the incidence of genital warts and the cost of illness in Germany: a cross-sectional study. BMC Infect Dis.

[15] Hoy T, Singhal PK, Willey VJ, Insinga RP (2009). Assessing incidence and economic burden of genital warts with data from a US commercially insured population. Curr Med Res Opin.

[16] Pirotta M, Stein AN, Conway EL, Harrison C, Britt H, Garland S (2010). Genital warts incidence and healthcare resource utilisation in Australia. Sex Transm Infect.

[17] Kulmala SM, Shabalova IP, Petrovitchev N (2007). Prevalence of the most common high-risk HPV genotypes among women in three new independent states of the former Soviet Union. J Med Virol.

[18] Shipitsyna E, Zolotoverkhaya E, Kuevda D (2011). Prevalence of high-risk human papillomavirus types and cervical squamous intraepithelial lesions in women over 30 years of age in St. Petersburg, Russia. Cancer Epidemiol.

[19] (2013). World Medical Association Declaration of Helsinki: ethical principles for medical research involving human subjects. JAMA.

[20] Vora R, Anjaneyan G, Doctor C, Gupta R (2011). Clinico-epidemiological study of sexually transmitted infections in males at a rural-based tertiary care center. Indian J Sex Transm Dis.

[21] Hoy T, Singhal PK, Willey VJ, Insinga RP (2009). Assessing incidence and economic burden of genital warts with data from a US commercially insured population. Curr Med Res Opin.

[22] Dinh TH, Sternberg M, Dunne EF, Markowitz LE (2008). Genital warts among 18- to 59-year-olds in the United States, national health and nutrition examination survey, 1999–2004. Sex Transm Dis.

[23] Lan CW, Scott-Sheldon LAJ, Carey KB, Johnson BT, Carey MP (2014). Alcohol and sexual risk reduction interventions among people living in Russia: a systematic review and meta-analysis. AIDS Behav.

[24] Kjaer SK, Tran TN, Sparen P (2007). The burden of genital warts: a study of nearly 70,000 women from the general female population in the 4 Nordic countries. J Infect Dis.

[25] Klavs I, Grgic-Vitek M (2008). The burden of genital warts in Slovenia: results from a national probability sample survey. Euro Surveill.

[26] Brotherton JM, Heywood A, Heley S (2009). The incidence of genital warts in Australian women prior to the national vaccination program. Sex Health.

[27] Fenton KA, Korovessis C, Johnson AM (2001). Sexual behaviour in Britain: reported sexually transmitted infections and prevalent genital Chlamydia trachomatis infection. Lancet.

[28] Pirotta M, Ung L, Stein A (2009). The psychosocial burden of human papillomavirus related disease and screening interventions. Sex Transm Infect.

[29] Marra F, Ogilvie G, Colley L (2009). Epidemiology and costs associated with genital warts in Canada. Sex Transm Infect.

[30] Kodner CM, Nasraty S (2004). Management of genital warts. Am Fam Physician.

[31] Harrison C, Britt H, Garland S, Conway L (2014). Decreased management of genital warts in young women in Australian general practice post introduction of national HPV vaccination program: results from a nationally representative cross-sectional general practice study. PLoS One.

[32] Arbyn M, Raifu AO, Weiderpass E, Bray F, Anttila A (2009). Trends of cervical cancer mortality in the member states of the European Union. Eur J Cancer.

