# Supplementary material for: Estimating the Burden of Illness Related to Genital Warts in Russia: A Cross-Sectional Study
Source: J Health Econ Outcomes Res. 2020 Oct 7;7(2):182–8. doi: 10.36469/jheor.2020.17246 (PMC7549541; doi:10.36469/jheor.2020.17246)
Supplement: Supplementary file 1 [file jheor-7-2-17246-s01.pdf]

Journal of Health Economics and Outcomes Research

Supplementary Online Material

Prilepskaya VN, Gomberg M, Kothari S, Yee K, Kulkarni A, Garland SM, Giuliano AR. Estimating the burden of illness related to genital warts in Russia: a rross-sectional study. *JHEOR*. 2020;7(2):182-188.
doi:10.36469/jheor.2020.17246

Referral Patterns, Health Care Resource Use

A total of 126 physicians completed the 30-minute survey, including 37 OB/GYN, 48 URO, and 41 DERM.

Male patients. Among the 89 physicians who completed the 30-minute survey and saw male patients, of the last 20 male GW patients seen, an average of 72.9% consulted with the participating physician directly and 27.1% were referred by another physician (Table S1). Among male patients, an average of 86.0% were treated by the participating physician directly, 11.5% were referred to another physician, 2.5% were first treated and then referred, and 0.1% of patients were left untreated or not referred.

The primary diagnostic technique utilized was clinical examination by visual examination (98%; Figure S1a). Seventy-eight physicians reported using HPV polymerase chain reaction to diagnose GW patients, and 71.4% of all patients received the test on average. Six physicians (1 URO, 5 DERM) reported using other diagnostic tools as well as performing full sexually-transmitted viral infection screens (including bacterioscopy, enzyme immunoassay, and serology for human immunodeficiency virus [HIV], hepatitis B virus carriage, hepatitis C, and syphilis tests) for 91.7% of their male patients.

The use of in-office treatment or procedures was also reported by participating physicians based on the last 20 male GW patients seen (Figure S2a). Results suggest that 80 out of 87 physicians provided a basic patient visit and 88.5% of all patients received treatment at that visit. Sixty-two physicians used electrosurgery for patient cases, including URO (54.3%) and DERM (26.2%). Thirty physicians, including URO (34.2%) and DERM (65.0%) of patients, used other treatments and procedures, including pheresolum (a solution used for radio wave surgery and the treatment of skin lesions like warts) and solcoderm (a solution which, in the past, was considered suitable for the treatment of malignant and benign skin lesions.)

Topical medication use for in-office treatment, based on the last 20 male GW patients seen, is presented in Figure S3a. Out of 87 physicians, 42 reported administering podofilox for male patients, and an average of 16.7% of all patients received this treatment. Twenty-five physicians reported using other topical medications for in-office treatment (cryopharma spray, curiosin, cycloferon, levomekol, panavir, pheresolum, pimafucort, rebif, resorcinol, solcoderm, triderm, ferrovir solution, lactic acid, salicylic acid) for 36.6% of male patients.

Prescribed at-home topical medication treatment use is shown in Figure S4a. Out of 87 physicians, 38 reported prescribing podofilox for male patients (average of 20.2% of all male patients). Thirty-seven physicians reported prescribing other topical medications for at-home treatment (5-fluorouracil, Amixin, Baneocin, curiosin, cycloferon, Ferezol, genferon, glycyrrhizic acid, Groprinosin, indinol, isoprinosine, levomekol, panavir, pimafucort, polyoxidonium, potassium permanganate, resorcinol, silver nitrate solution, solcoderm, synthomycine, triderm, Viferon, Zovirax, glycyrrhetinic acid, interferon, lactic acid, salicylic acid) for 38.0% of male patients.

This is an open-access article distributed under the terms of the Creative Commons Attribution 4.0 International License (CCBY-4.0). View this license's legal deed at http://creativecommons.org/licenses/by/4.0 and legal code at http://creativecommons.org/licenses/by/4.0/legalcode for more information.

Female patients. Of the 118 physicians who completed the 30-minute survey and saw female patients, an average of 63.1% of the last 20 patients seen consulted the physician directly, while 36.1% were referred by another doctor. Of those last 20 female patients seen, an average of 65.3% were treated by the participating physician directly, 28.7% were referred to another physician, 4.3% were first treated and then referred, and 1.0% were left untreated or not referred. URO referred more of their female patients (43.0%) to another physician compared to other specialists (Table S1).

In female GW patients, the diagnostic tools and techniques used most frequently by physicians to diagnose GW were visual examination (92.7%; Figure S1b). Ninety-two physicians reported using the HPV polymerase chain reaction test to diagnose GW, and 64.3% of all patients received the test on average. Sixty-three physicians reported using Papanicolaou (Pap) tests with 91.7% (OB/GYN), 44.5% (DERM), and 12.0% (URO) of patients. Seven physicians reported using other tools and techniques (bacterioscopy, enzyme immunoassay and HIV, hepatitis B, hepatitis C and syphilis tests, colpocytogram) for 92.9% of female patients.

The use of in-office treatment or procedures was also reported by participating physicians based on the last 20 female GW patients seen (Figure S2b). Of the physicians surveyed, 95 out of 120 completed a basic visit, and 77.4% of all patients received this treatment. Fifty-two physicians used electrosurgery for in-office treatment, and 22.5% of all female patients received the procedure. Forty-six physicians used other treatments and procedures—including Condyline, laser surgery, radio wave surgery, and solcoderm—for 48.5% of female patients.

Topical medication use for in-office treatment is displayed in Figure S3b, based on the last 20 female GW patients seen. For female patients, 44 out of 119 physicians reported using other topical medications for in-office treatment (cryopharma spray, curiosin, cycloferon, epigen, genferon levomekol, panavir, pheresolum, pimafucort, rebif, resorcinol, solcoderm, Tricresolum, triderm, lactic acid, salicylic acid) for 35.9% of patients. Forty-three physicians reported using podofilox and 13.4% of all female patients received this treatment.

Prescribed topical medications for at-home treatment (Figure S4b) followed a similar pattern as in-office treatment use. Other topical medications for at-home treatment (allomedin, Baneocin, cryopharma, curiosin, cycloferon, epigen, Ferezol, genferon, glycyrrhizic acid, Groprinosin, immunomodulators, indinol [vaginal suppositories], isoprinosine, Lavomax, levomekol, panavir, pimafucort, solcoderm, synthomycine, triderm, Viferon, interferon, lactic acid) were reported in 50.3% of female patients. Twenty-eight physicians reported using podofilox and 9.7% of all female patients received this treatment.

[1] Grunwald MH, Gat A, Amichai B. Pseudomelanoma after solcoderm treatment. Melanoma Res. 2006;16(5):459–460.

Table S1. Referral and Treatment Status of the Last 20 GW Patients (Participating Physicians for 30-Minute Survey)

	Specialty Group			
	**OB/GYN (N=37)**	**URO (N=48)**	**DERM (N=41)**	**Overall (N=126)**
Number of physicians who saw male GW patients	0	48	41	89
**Male Patients**				
% seen by participating physician directly, without referral	.	77.1	68.0	72.9
% referred from another physician	.	22.9	32.0	27.1
% treated by the participating physician	.	89.7	81.6	86.0
% referred to another physician for treatment	.	8.3	15.2	11.5
% treated by participating physician then referred to another	.	1.9	3.2	2.5
% neither treated by participating physician nor referred to another	.	0.1	0.0	0.1
Number of physicians who saw female GW patients	37	43	38	118
**Female Patients**				
% seen by participating physician directly, without referral	74.9	60.5	54.6	63.1
% referred from another physician	25.1	37.4	45.4	36.1
% treated by the participating physician	73.5	51.4	72.9	65.3
% referred to another physician for treatment	17.8	43.0	23.0	28.7
% treated by participating physician then referred to another	5.9	3.5	3.7	4.3
% neither treated by participating physician nor referred to another	2.7	0.0	0.4	1.0

Abbreviations: DERM=dermatologist; GW, genital warts; OB/GYN, obstetrician/gynecologist; URO, urologist.
Percentages calculated as % of last 20 GW patients seen by each physician, summarized over physicians in specialty group. There was one physician who provided data for <20 patients.

Figure S1a. Diagnostic Tools and Techniques Used for Male GW Patients (Participating Physicians for 30-minute Survey)

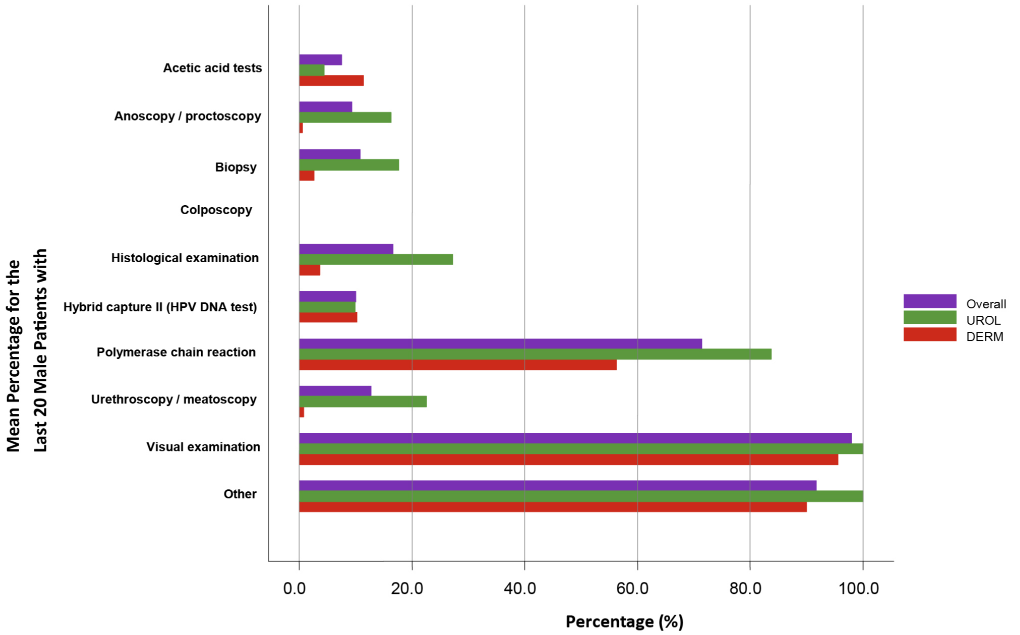

Abbreviations: DERM, dermatologist; DNA, deoxyribonucleic acid; GW, genital warts; URO: urologist.
Other tools and techniques included bacterioscopy, enzyme immunoassay and HIV, hepatitis B, hepatitis C, and syphilis tests.

Figure S1b. Diagnostic Tools and Techniques Used for Female GW Patients (Evaluable Physicians for 30-minute Survey)

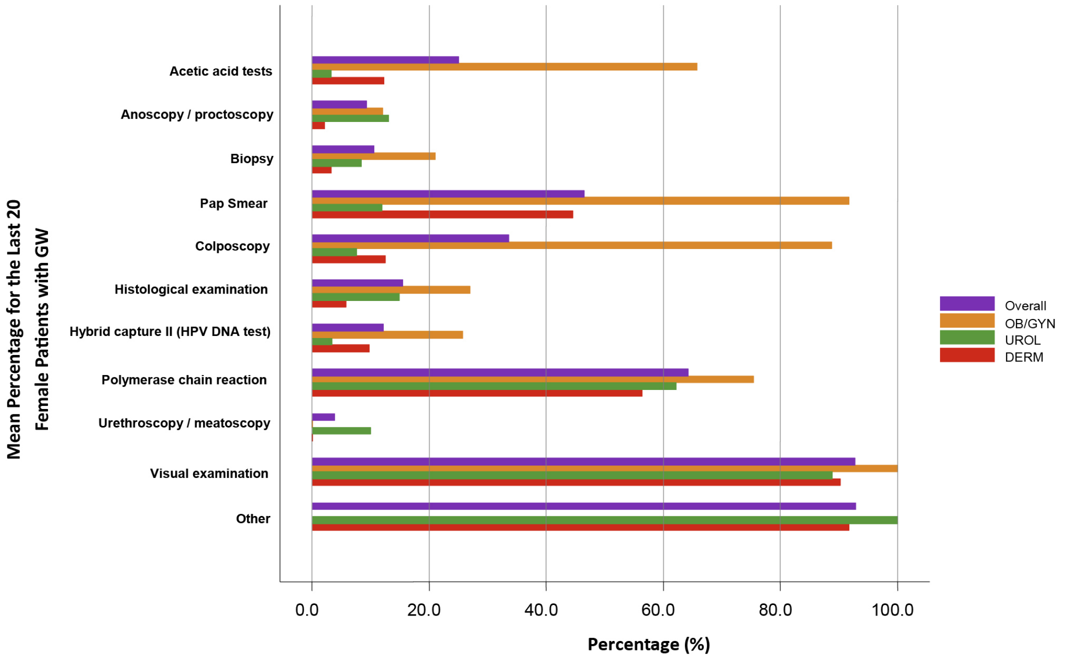

Abbreviations: DERM, dermatologist; DNA, deoxyribonucleic acid; GW, genital warts; OB/GYN, obstetrician/gynecologist; Pap, Papanicolaou test; UROL, urologist.
Other tools and techniques included bacterioscopy, enzyme immunoassay and HIV, hepatitis B, hepatitis C, syphilis tests, and colpocytogram.

Figure S2a. In-office Treatments or Procedures Used for Male GW Patients (Participating Physicians for 30-minute Survey)

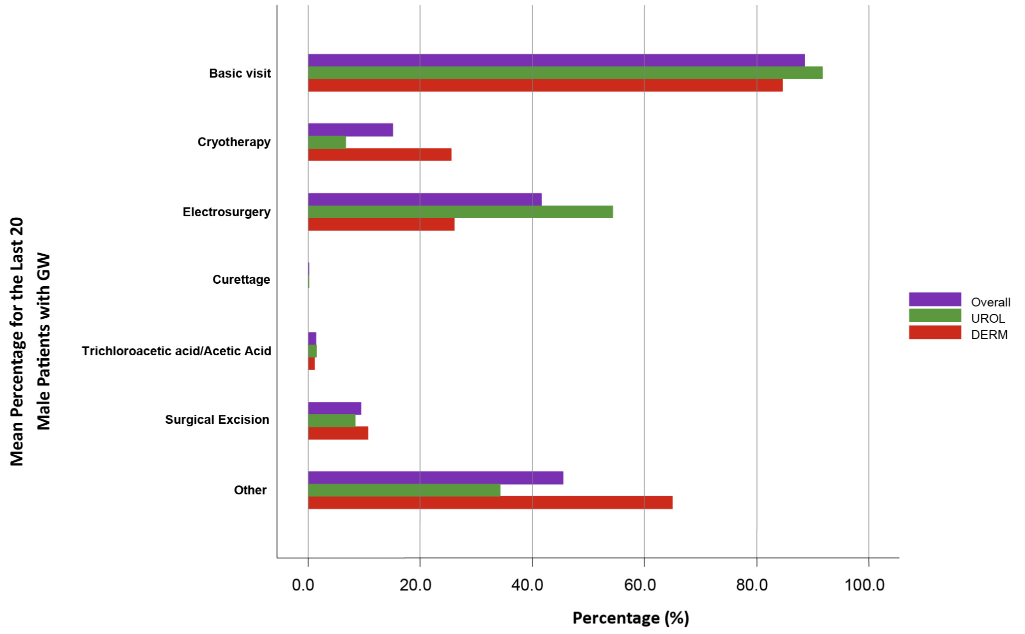

Abbreviations: DERM, dermatologist; GW, genital warts; URO, urologist.
Other diagnostic treatments and procedures included pheresolum, radio wave surgery, and solcoderm.

Figure S2b. In-office Treatments or Procedures Used for Female GW Patients (Participating Physicians for 30-minute Survey)

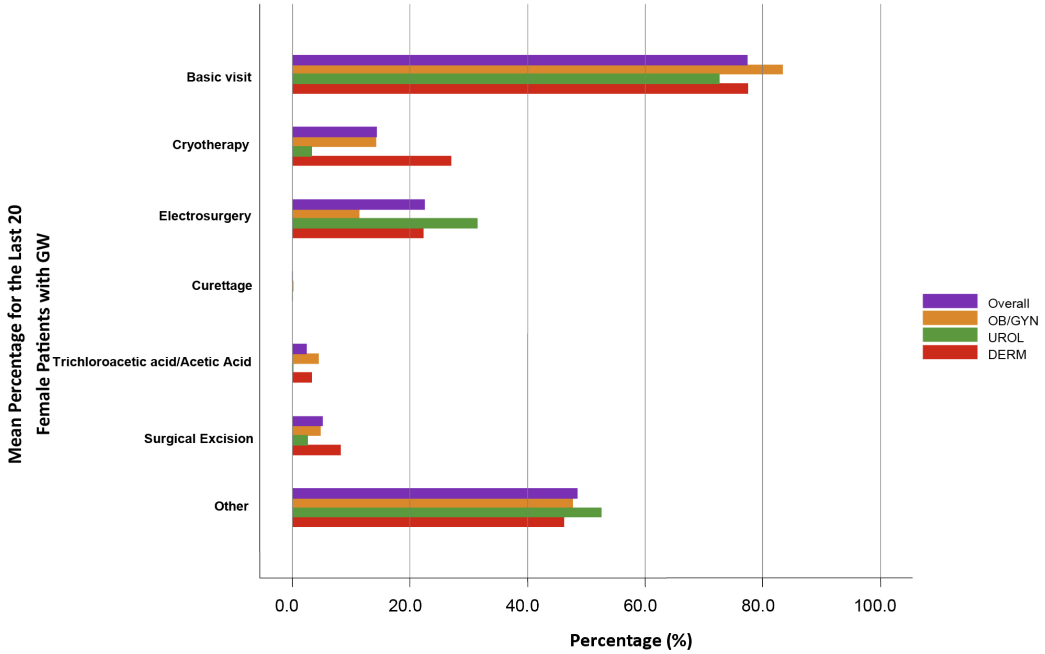

Abbreviations: DERM, dermatologist; GW, genital warts; OB/GYN, obstetrician/gynecologist; URO, urologist.
Other treatments and procedures included Condyline, laser surgery, radio wave surgery, and solcoderm.

Figure S3a. Topical Medications Used for In-office Treatment for Male GW Patients (Evaluable Physicians for 30-minute Survey)

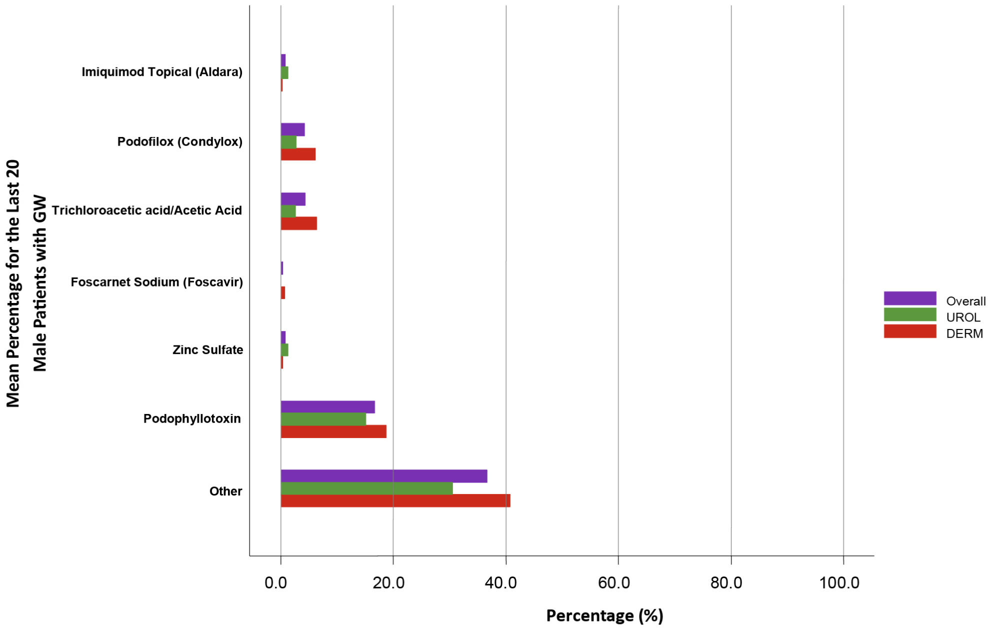

Abbreviations: DERM, dermatologist; GW, genital warts; URO, urologist.
Other topical medications used for in-office treatment included cryopharma spray, curiosin, cycloferon, levomekol, panavir, pheresolum, pimafucort, rebif, resorcinol, solcoderm, triderm, ferrovir (solution), lactic acid, and salicylic acid.

Figure S3b. Topical Medications Used for In-office Treatment for Female GW Patients (Evaluable Physicians for 30-minute Survey)

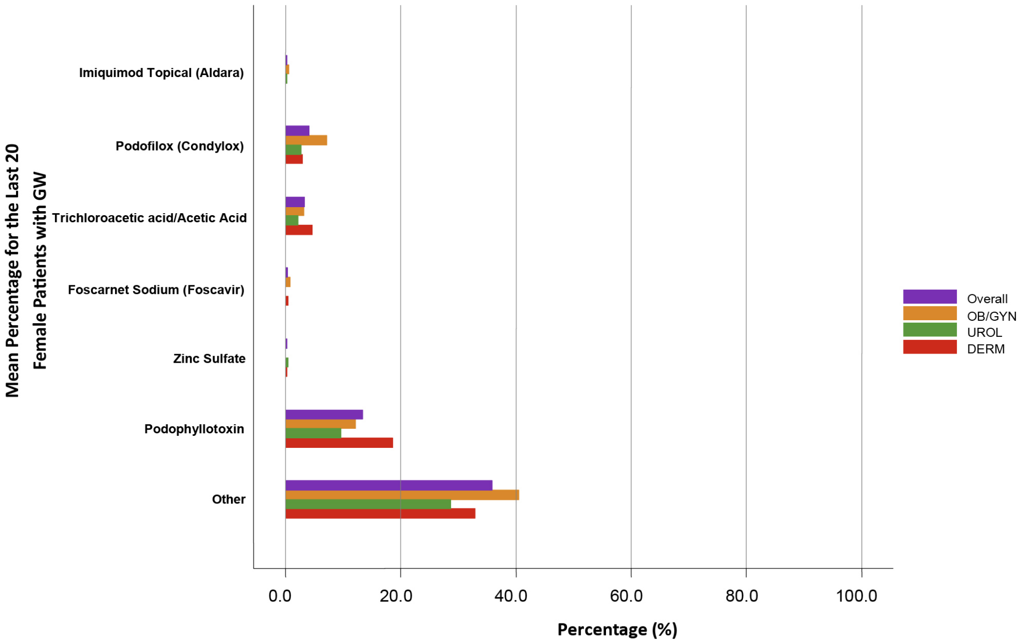

Abbreviations: DERM, dermatologist; GW, genital warts; OB/GYN, obstetrician/gynecologist; URO, urologist.
Other topical medications used for in-office treatment included cryopharma spray, curiosin, cycloferon, epigen, genferon levomekol, panavir, pheresolum, pimafucort, rebif, resorcinol, solcoderm, tricresolum, triderm, lactic acid, and salicylic acid.

Figure S4a. Topical Medications Used for At-home Treatment for Male GW Patients (Evaluable Physicians for 30-minute Survey)

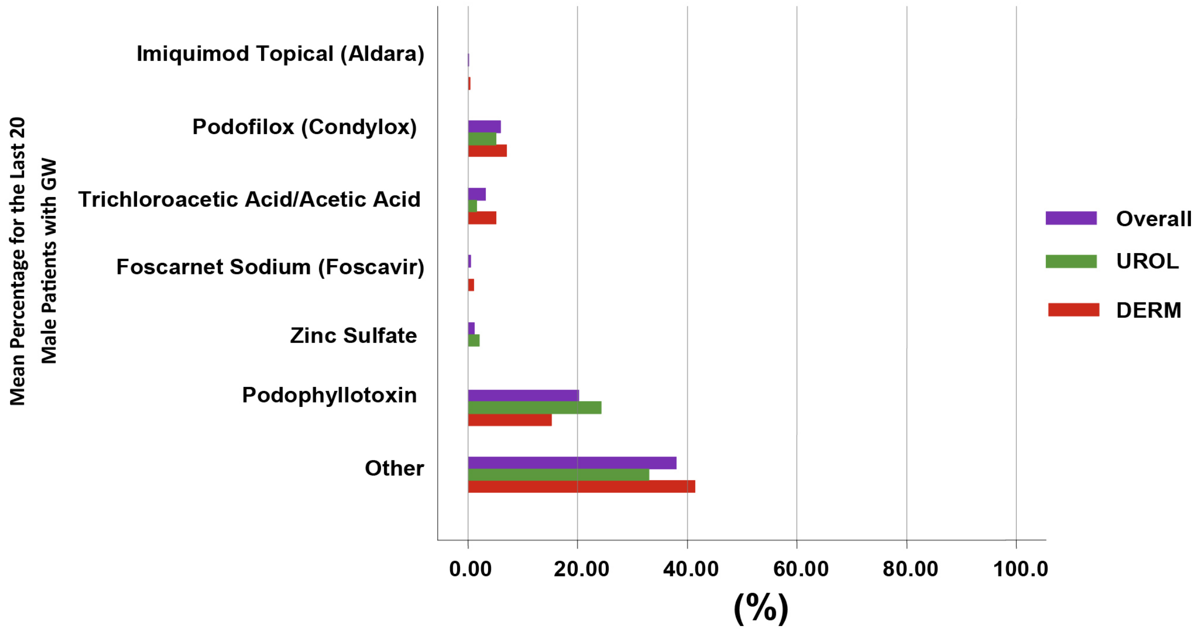

Abbreviation: GW, genital warts.

Figure S4b. Topical Medications Used for At-home Treatment for Female GW Patients (Evaluable Physicians for 30-minute Survey)

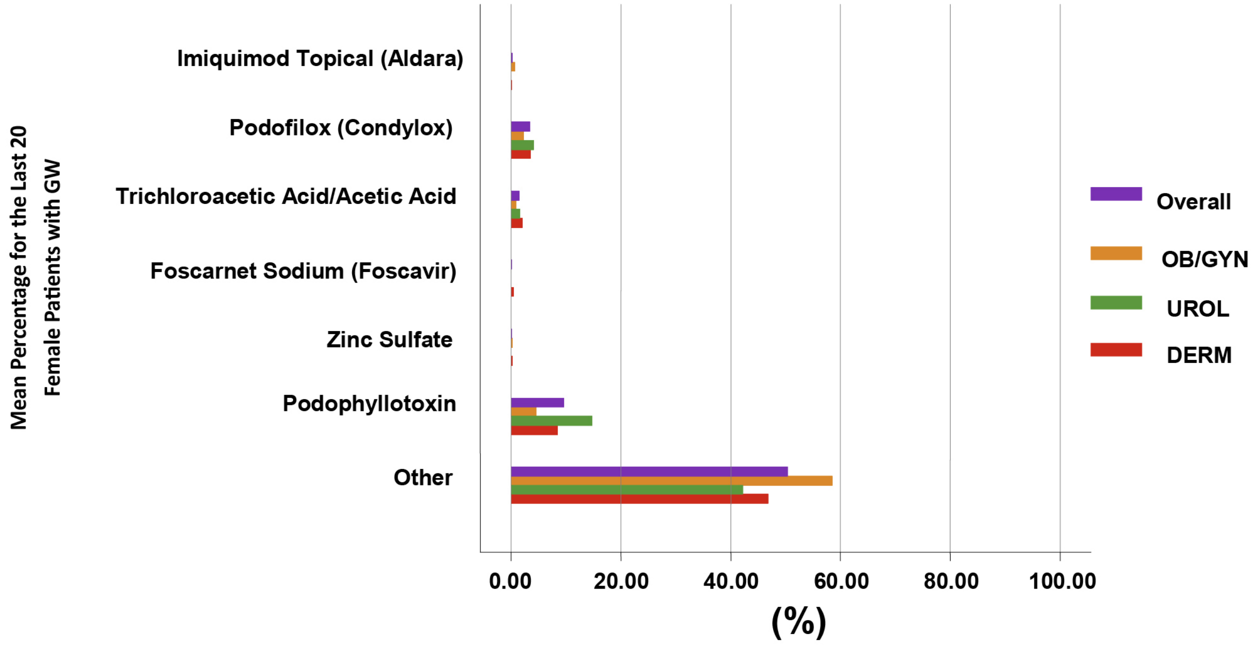

Abbreviation: GW, genital warts.
